# Supplementary material for: Are carbon emissions trading and green financial instruments synergistic? -Comprehensive quantitative research based on content analysis
Source: PLoS One. 2024 Mar 7;19(3):e0298601. doi: 10.1371/journal.pone.0298601 (PMC10919601; doi:10.1371/journal.pone.0298601)
Supplement: S1 Data — (ZIP) [file pone.0298601.s001.zip › Data/地级市Z_F工作报告.zip!/▒▒╛⌐/└┤╫╘╣1⁄2╓┌║┼í╛┬φ┐╦╩2╛▌═°í┐_2013.docx]

——2013年1月22日在北京市第十四届人民代表大会第一次会议上

北京市代市长 王安顺

　　各位代表：

　　现在，我代表北京市人民政府，向大会报告本届政府过去五年的工作，对今年工作提出建议，请予审议，请市政协各位委员提出意见。

　　一、过去五年工作回顾

　　2008年以来的五年，是首都发展进程中具有重要历史意义、极不平凡的五年。在党中央、国务院和中共北京市委坚强领导下，全市人民认真贯彻党的十七大精神，深入实施“人文北京、科技北京、绿色北京”战略，总结提炼了“北京精神”，胜利完成“十一五”规划，全面实施“十二五”规划，首都改革开放和现代化建设取得令人瞩目的成就。

　　(一)圆满完成奥运会和国庆60周年重大活动筹办任务。全面践行“绿色奥运、科技奥运、人文奥运”理念，依靠举国体制优势，依靠人民群众参与奉献，成功举办了一届有特色、高水平的奥运会、残奥会，为圆中华民族百年奥运梦想作出了应有贡献。坚持首善标准，全力筹办新中国成立60周年庆祝活动，实现了“隆重、喜庆、节俭、祥和”的目标。奥运、国庆两大活动的圆满成功，有力提升了城市文明程度和服务管理水平，实现了“新北京、新奥运”战略构想，留下了弥足珍贵的物质精神财富，推动首都发展进入了新阶段。

　　(二)首都经济迈上新台阶。认真贯彻落实中央决策部署，积极应对国际金融危机影响，及时制定实施一系列稳增长、惠民生的具体措施，克服困难，深化改革，经受住了复杂形势的严峻考验，保持了经济平稳较快发展。初步核算，2012年全市地区生产总值达到1.78万亿元，五年平均增长9.1%；人均地区生产总值提高到13797美元，达到中上等收入国家水平。全社会固定资产投资达到6462.8亿元，年均增长14.8%；社会消费品零售额达到7702.8亿元，是2007年的2倍。地方公共财政预算收入达到3314.9亿元，年均增长17.3%。

　　(三)经济结构加快调整转型。产业结构进一步优化，生产性服务业、文化创意产业比重分别比2007年提高了5.6个和2.1个百分点，服务业比重由73.5%提高到76.4%，高技术制造业较快发展，都市型现代农业体系初步建立，首都经济的特征更加彰显。中关村国家自主创新示范区建设取得重大进展，实现企业总收入2.4万亿元，是2007年的2.7倍。全社会研发强度达到5.8%，技术合同成交额占全国的38.2%。加快重点功能区建设发展，六大高端功能区占全市经济比重超过40%。突出抓好重点领域节能减排，完成首钢石景山厂区涉钢产业搬迁改造，累计关闭446家高污染、高耗能、高耗水企业，告别了近千年的小煤窑采矿史，单位地区生产总值能耗、水耗分别累计下降25%以上和32%，节能减排走在全国前列，综合发展指数连续多年排名第一。

　　(四)城乡区域发展更趋协调。不断完善强农惠农富农政策体系，完成农村集体经济产权制度改革和集体林权制度改革主体任务，12万农民在护林、管水等公益性岗位就业。市政府投资投向郊区比重连续八年超过50%，高速公路通达每个区县，郊区新城全部集中供暖，基本实现村村通油路、通公交。累计投资280亿元，完成新农村建设“5+3”工程，农民生产生活条件明显改善。城南行动三年计划、城乡结合部50个重点村城市化改造成效显著，通州等重点新城建设提速，西部地区转型发展步伐加快，生态涵养发展区活力增强，为实现科学发展打下了坚实基础。

　　(五)城市建设管理不断加强。五年累计投资近1900亿元，建成轨道交通300公里，通车总里程达到442公里。全面实施建、管、限综合治堵措施，在机动车保有量增加210万辆的情况下，市区交通运行保持基本平稳。南水北调中线北京段、城市电网改造等一批重大项目建成使用，资源能源保障能力明显增强。加强污染全方位治理，空气中主要污染物浓度平均下降29.3%，生活垃圾焚烧和生化处理比重由10%提高到50%，污水处理率、再生水利用率分别达到83%和61%。大力实施平原地区百万亩造林绿化，建成开放一批森林公园和郊野公园，完成京津风沙源治理、三北防护林工程阶段任务，全市森林覆盖率达到38.6%。实施200项重点文物保护工程，前门、什刹海等地区保护更新有力推进，展示了历史风貌与现代生活有机融合的新形象。

　　(六)民生保障水平明显提高。城乡居民收入分别达到36469元和16476元，年均实际增长7.5%和8.7%，农民收入增速连续四年快于城镇居民。实施学前教育、中小学、市属高校建设三个“三年行动计划”，累计增加幼儿园入园名额5.7万个，完成了中小学的校舍安全工程，高等教育、职业教育取得长足进步。医疗机构管办分开、医药分开改革实现突破，医药卫生体制改革三年任务顺利完成，医疗卫生服务不断改善。完成文艺院团转企改制任务，成立六大文化联盟，建立了四级公共文化服务体系。竞技体育取得良好成绩，全民健身运动蓬勃开展。城乡一体的促进就业格局基本形成，城镇累计新增就业217.6万人，登记失业率始终控制在2%以内。社保相关待遇标准五年提高六次，各项标准均提高50%以上。全力推进“大民政”建设，实施居家养老助残政策和老年人优待办法，推动社会福利向适度普惠型转变。坚持房地产市场调控，促进房地产结构调整，加强老旧小区综合整治，累计提供各类保障房80万套。经过全市上下共同努力，实现了每个困难家庭至少有一人就业，实现了养老保障制度城乡一体化和医疗保险制度城乡全覆盖，实现了“村村有书屋”、广播电视“户户通”，实现了廉租房应保尽保。人民群众更好地享受到改革发展成果，生活水平不断提高。

　　(七)社会建设和服务管理开创新局面。制定实施全面推进社会建设的意见和规划，率先形成社会建设四级工作网络。开展社会组织直接登记试点，建立政府购买社会组织服务机制，构建“枢纽型”社会组织工作体系。加强社会工作者和志愿者队伍建设，选聘7067名大学生到社区工作。第六次人口普查全面完成，人口计划生育工作取得新成绩。民族、宗教工作进一步加强。“五五”普法任务圆满完成，“六五”普法扎实推进。双拥共建和国防后备力量建设取得新进展，驻京部队在首都现代化建设和重大活动中作出了重要贡献。强化综合监管，严厉打击非法违法生产经营建设、治理纠正违规违章行为，安全生产事故起数比2007年下降27.4%。

　　实施重大决策社会稳定风险评估，市、区县、街道乡镇三级风险评估体系基本建立。深入开展领导干部接访、下访和社会矛盾排查化解，建立多元化矛盾调解体系，推进人民调解、行政调解、司法调解有效衔接，连续五年实现信访总量、集体访、联名信三下降。加强和创新社会管理，网格化服务管理体系建设全面推开，村庄社区化管理分类推进。深入推进“平安北京”建设，完善社会治安防控体系和治安重点地区排查整治机制，加强流动人口、特殊人群服务管理，创新警务模式，首都安全稳定大局进一步巩固。

　　(八)改革开放实现新突破。完成首都功能核心区行政区划调整，推动大兴区和北京经济技术开发区行政资源整合。加大国有企业改革重组力度，建立完善国有资本运营平台，市属企业营业收入和利润比2007年分别增长97%和88%。制定实施一系列支持民间投资、中小微企业发展的意见。完善市与区县分税制财政管理体制，营业税改征增值税试点顺利推进。规范发展产权、林权、技术等要素市场，三农、中小企业金融服务体系初步形成。精心打造“京交会”品牌，积极构建口岸经济体系，国际商贸中心建设取得阶段性成果。地区进出口总额达到4079.2亿美元、年均增长16.1%，实际利用外资突破80亿美元。发起成立世界旅游城市联合会，制定实施促进旅游业发展意见，年入境游客超过500万人次。深化对外务实交流合作，港澳台侨工作扎实推进。高标准完成什邡、玉树灾后援建任务，对口支援和区域合作成效显著。

　　(九)政府自身建设取得新进展。全面执行市人大及其常委会决议和决定，坚持重大事项报告制度，自觉接受人大工作监督、法律监督和人民政协民主监督，积极听取各民主党派、工商联、无党派人士和人民团体意见。五年来，市政府共办理市人大代表议案17项、建议7228件，办理市政协建议案33件、提案6305件，提请市人大常委会审议地方性法规草案25项，制定修改政府规章41项。深化行政审批制度改革，市级部门取消审批事项64项、下放审批权限126项。完成市与区县政府、乡镇机构改革任务。建立覆盖市、区县两级的“三效一创”绩效管理体系，政府绩效管理工作全面推开。加强廉政风险防控管理，强化行政监察和审计监督，严肃查处各类违纪违法案件。

　　回顾过去五年，我们深切感受到，这是举全市之力，办成大事、办好喜事，推动首都科学发展取得重大进展的五年；是转变经济发展方式迈出坚实步伐，综合经济实力显著增强的五年；是城乡面貌焕然一新，城市服务管理水平明显提升的五年；是保障和改善民生力度最大，人民群众得到更多实惠的五年。五年的成就来之不易，这是党中央、国务院和中共北京市委坚强领导，全市人民团结拼搏的结果，是全国各族人民大力帮助，社会各界关心支持的结果。在这里，我代表北京市人民政府，向全市各族人民，向人大代表、政协委员，向各民主党派、各人民团体和各界人士，向中央各部门、各兄弟省区市，向驻京解放军和武警部队官兵，向所有关心支持首都建设的港澳台同胞、海外侨胞和国际友人，表示崇高的敬意和衷心的感谢！

　　各位代表，五年的团结奋斗，深化了我们对首都工作和首都建设发展的认识，主要体会有：

　　一是必须坚持科学发展。要更加自觉地把解放和发展生产力作为根本任务，深入推进经济结构战略性调整，加快转变经济发展方式，大力发展首都经济，建立创新驱动发展模式，实现以人为本、全面协调可持续发展。

　　二是必须坚持“四个服务”。要认真贯彻落实中央对北京市工作的一系列指示精神，紧紧围绕首都城市性质和职责，不断完善城市功能，以服务促进发展，以发展优化服务，在服务国家大局中提升首都工作水平。

　　三是必须坚持民生优先。要把保障和改善民生作为建设发展的出发点和落脚点，深入推进基本公共服务均等化，切实解决人民群众最关心、最直接、最现实的利益问题，坚决维护首都社会和谐稳定，不断提高发展的包容性，让人民生活更加幸福美好。

　　四是必须坚持统筹协调。要遵循首都城市发展规律，发挥规划引导发展、规范发展、服务发展的作用，努力实现经济社会发展与城市功能定位相协调，与人口资源环境承载能力相适应，与更好地满足人民群众日益增长的物质文化需求相统一。

　　五是必须坚持改革开放。要系统谋划、凝聚共识，不失时机地推进重要领域改革，做到改革不停顿、开放不止步，在改革开放中促进发展、改善民生、破解难题，加快构建有利于科学发展的体制机制。

　　六是必须坚持群众路线。要尊重人民主体地位，紧紧依靠人民群众，发挥人民群众的首创精神。坚持问计于民，从人民的伟大实践中汲取智慧和力量；坚持问需于民，多办让人民群众满意的好事实事，与人民群众同甘共苦，共同推进首都现代化建设各项事业。

　　我们清醒地认识到，北京正处在转型发展的关键时期，发展中不平衡、不协调、不可持续问题依然突出，深化改革开放和转变经济发展方式任务艰巨；人口资源环境矛盾明显，优化城市空间和产业布局、调整经济结构、调控人口规模、治理交通拥堵、改善空气质量任重道远；统筹做好新阶段特大型城市规划、建设、运行、服务、管理工作需要进一步加大力度；创新驱动、内生增长的动力仍显不足，推动科技创新、文化创新，培育新的经济增长点还需下更大功夫；创新社会管理、解决好群众最关心的民生问题还需付出更多努力；政府职能转变还不到位，一些工作人员责任意识、服务意识不够，作风建设和廉政建设需要进一步加强。我们一定牢记职责使命，加倍努力工作，采取切实有效的措施解决好这些问题，决不辜负党中央的期望，决不辜负人大代表、政协委员的信任，决不辜负全市人民的重托。

　　二、今后五年的奋斗目标和2013年工作任务的建议

　　中国共产党第十八次全国代表大会胜利召开，描绘了全面建成小康社会、加快推进社会主义现代化、夺取中国特色社会主义新胜利的宏伟蓝图，为在新的起点上推动首都科学发展、促进社会和谐指明了方向。我们要深入贯彻落实党的十八大精神，高举中国特色社会主义伟大旗帜，紧紧抓住和用好可以大有作为的重要战略机遇期，深刻认识和把握发展的阶段性特征，深入实施“人文北京、科技北京、绿色北京”战略，全面深化改革开放，奋力推进首都经济、政治、文化、社会和生态文明建设，向着建设中国特色世界城市迈出更大步伐。

　　按照中共北京市第十一次代表大会的部署，今后五年，要在现有良好基础上实现新的发展要求。

　　——更加注重提高发展质量。转变经济发展方式取得重大进展，首都经济保持持续平稳增长，人均地区生产总值达到2万美元，到2020年实现地区生产总值比2010年翻一番，率先形成科技创新、文化创新“双轮驱动”的发展格局，初步建成有世界影响力的科技文化创新之城。大力发展绿色经济，产业结构进一步优化升级，服务业比重达到78%以上，“北京服务”、“北京创造”品牌的影响力明显增强。

　　——更加注重完善城市功能。率先形成城乡发展一体化新格局，新农村建设持续推进。优化城市空间布局，构建“六高四新”高端产业发展格局，区域发展的协调性不断增强。公共交通出行比例达到50%以上，交通拥堵得到有效治理。“智慧城市”的基本框架初步形成，城市运行的精细化、信息化、便利化水平明显提高，应急体系更加完善，城市服务保障能力显著增强。

　　——更加注重繁荣首都文化。全面践行“北京精神”，市民文明素质和城市文明程度显著提升，历史文化资源得到有效保护、传承和利用，文化事业和文化创意产业健康快速发展，公共文化服务体系更加完善，文化创新充满活力，全国文化中心的示范作用得到充分发挥，努力建设中国特色社会主义先进文化之都。

　　——更加注重促进社会和谐。社会服务管理体系更加完善，人口服务管理更加科学有序，社会矛盾化解体制机制更加健全。依法治国基本方略更加深入人心，社会生活制度化、法制化水平明显提高，社会公平正义得到有效维护。公民有序参与社会管理，群众利益表达渠道更加畅通。

　　——更加注重生态文明建设。资源节约型、环境友好型社会建设取得重大进展，完成平原地区100万亩造林任务，全市森林覆盖率达到40%以上，主要污染物排放总量持续削减。生态文明制度进一步完善，全社会节约意识、环保意识、生态意识明显增强，形成合理消费的社会风尚。

　　——更加注重深化改革开放。有利于推动科学发展、加快经济发展方式转变和自主创新的体制机制与制度进一步完善，重要领域和关键环节改革取得明显进展。政府职能加快转变，市场配置资源的基础性作用得到充分发挥。国际化水平进一步提高，聚集国际高端要素的能力不断增强，努力建设国际活动聚集之都、世界高端企业总部聚集之都、世界高端人才聚集之都。

　　——更加注重改善市民福祉。就业更加充分，城乡居民人均收入到2020年比2010年翻一番，城乡收入差距逐步缩小，中低收入群众的收入水平明显提高。完成5000万平方米老旧小区综合整治和100万套保障房建设任务。基本公共服务更趋均等化，非基本公共服务更加丰富。基本实现义务教育资源均等化，基本满足适龄儿童入园需求，率先实现教育现代化，覆盖城乡的社会保障制度更加完善，努力建设和谐宜居之都。

　　今年，是全面贯彻落实党的十八大精神的开局之年，是实施“十二五”规划承前启后的关键一年。政府工作的总体要求是：深入学习贯彻党的十八大精神，坚持以邓小平理论、“三个代表”重要思想、科学发展观为指导，紧紧围绕主题主线，以提高经济增长质量和效益为中心，稳中求进，开拓创新，深化改革开放，强化创新驱动，加大产业结构优化升级力度，着力保障和改善民生，切实提高城市精细化管理水平，加快城乡一体化步伐，全力履行好“四个服务”职责，统筹推进首都经济、政治、文化、社会和生态文明建设，确保首都各项工作开好局、起好步。

　　今年全市经济社会发展主要预期目标是：地区生产总值增长8%左右；城镇登记失业率控制在2%左右；城乡居民收入实际增长7.5%左右；居民消费价格涨幅控制在4%左右；地方公共财政预算收入增长9%；单位地区生产总值能耗下降2%以上、二氧化碳排放量下降2.5%以上、水耗下降4%以上，主要污染物排放量均削减2%。

　　围绕实现今年经济社会发展目标，要着重抓好以下几个方面的工作：

　　(一)加快转变经济发展方式，促进首都经济持续健康发展

　　充分发挥首都资源优势、服务优势，创新体制机制，加快产业转型升级，调整优化需求结构，增强发展的平衡性、协调性和可持续性。

　　实施创新驱动发展战略。一是深化中关村示范区先行先试。认真落实国务院关于调整中关村空间规模和布局的批复精神，健全一区十六园管理体制，加强产业统筹规划。完善中关村创新平台，深入推进现有政策落实，积极争取新的先行先试政策，努力在市级相关政策和机制创新上取得突破。以中关村核心区为重点，打造科技金融功能区，加快建设国家科技金融创新中心。二是构建以企业为主体的技术创新体系。推动与中央单位、高校院所开展协同创新，支持企业建设研发机构、承接重大项目，培育一批成长快、潜力大的创新企业。三是加快推动重大科技成果产业化。统筹用好100亿元专项资金，更加注重引导放大作用和使用效益。坚持直接支持与提供市场并重，完善高技术产品采购机制，推进北斗导航、物联网等示范应用。四是加大人才特区建设力度。认真组织实施“千人计划”、“海聚工程”，继续推进中关村科学城、未来科技城建设，大力培养和引进高端技术研发人才、产业领军人才。实施知识产权、标准化和商标战略，加强知识产权保护，营造尊重知识、尊重创造的社会氛围。

　　培育产业发展新优势。认真抓好石景山、中关村现代服务业改革试点，制定加快流通产业发展实施方案，完善软件和集成电路产业发展鼓励政策，设立促进金融产业发展基金，积极发展多层次资本市场，巩固提升金融、信息、商务、科技等服务业发展水平。大力发展非基本公共服务，健全行业服务及准入标准，探索设立产业引导基金，鼓励社会资本进入养老、医疗、健康、培训等领域，加快市场化、产业化步伐。抓好国家旅游综合改革试点工作，推进旅游功能区和重大项目建设，打造“大旅游”产业链，推动旅游业成为重要支柱产业。坚持聚焦重点，推动制造业高端化发展，培育轨道交通、生物医药、集成电路、卫星应用等特色产业集群，力争在新一代信息技术、节能环保、新能源汽车、新材料等战略性新兴产业发展上取得突破。

　　巩固扩大内需。更好地发挥消费的基础作用和投资的关键作用，实现社会消费品零售额、固定资产投资分别增长11%和9%左右。出台促进消费、建设良好消费环境的意见，鼓励支持网络购物等新型业态发展，推动商贸服务转型升级，规范发展家政服务业，扩大文化、娱乐、培训、医疗保健等服务型消费规模。充分发挥72小时过境免签政策的作用，挖掘高端商务、会展、休闲度假等消费潜力，拓展外来消费。着力促进民生消费，推进家具以旧换新工作，启动15万个小微商户便民金融服务设施建设，延伸保障房入住等住房消费服务。进一步优化投资结构，提高民间投资比重。扎实推进在建、续建工程，加大改善民生、基础设施、生态环境、防灾减灾等领域投资力度。积极推动金融创新，规范一批承担重大建设任务的区县融资平台，做强做优轨道交通、水利工程等融资平台，有效拓展融资渠道。继续坚持房地产市场调控政策，加快建立和完善引导房地产业健康发展的长效机制。全力抓好重要商品的供应保障，着力降低流通成本，加强价格监测，整顿市场秩序，保持物价水平基本稳定。

　　优化高端产业发展布局。健全重大项目市级统筹机制，完善园区投入产出考核评价体系，推动各类功能区协调发展、特色发展。落实加快北京经济技术开发区发展的意见，强化金融街的国家金融中心功能，增强商务中心区的国际商务功能，提高临空经济区和奥林匹克中心区发展水平。加强规划统筹，突出功能定位，推进通州高端商务服务区、新首钢功能区、丽泽金融商务区、怀柔文化科技产业新区、海淀北部生态科技新区、北京科技商务区和区县各类功能区建设。

　　全力服务各类企业在京发展。加强与中央企业战略合作，抓好全过程服务，加快项目落地建设，吸引更多中央优质资源在京聚集，实现共赢发展。完善和延伸跨国公司总部政策，吸引国内外更多企业总部在京实体化发展，拓展总部经济发展领域。抓紧出台中小企业法实施细则，设立中小企业发展基金，发挥民间商会和行业协会作用，促进非公有制经济健康发展。完善统筹服务机制，及时帮助企业解决实际问题，为各类市场主体营造良好的发展环境。

　　(二)加强城市建设和管理，不断提升城市综合服务水平

　　深化系统研究，完善政策措施，积极探索创新，加快解决现阶段面临的突出矛盾和问题，全力推动城市发展转型。

　　科学治理交通拥堵。坚持标本兼治，落实交通综合治堵28项措施，学习借鉴国内外城市成功经验，研究制定新的治堵方案，做到年年有计划、年年有成效。一是加快发展轨道交通。优先加密建设中心城区线网，年内新开工建设3条线路。二是提升路网通行能力。认真做好兴延路、姚家园路等项目的前期工作，建成梅市口西延等重点道路，加快建设京张城际、京沈高铁、国道110二期等对外交通疏解通道。实施一批道路微循环改造工程，开工建设苹果园等交通枢纽，新建10处驻车换乘停车场。优化公交线网，新增公交快速线路50公里，增开社区与轨道交通、地面公交的通勤线路，公共交通出行比例达到46%。三是增强城市交通疏导能力。提高交通指挥信息发布平台效用，引导车辆均衡出行。全面摸排交通堵点，制定工程改造和一对一疏导方案，提高易堵路口和节点通行效率。四是加强停车服务与管理。完善土地、价格、补贴等政策，加快停车设施建设，新增居住区停车位2万个。充分挖掘社会资源，合理利用机关、学校、企业停车设施，推广错时停车和居住区停车自治管理。进一步控制小汽车过快增长。加大“黑车”整治力度，加强出租车运营管理。改善城市慢行系统，推广公共租赁自行车服务，倡导绿色交通、文明交通理念。

　　提高城市防灾减灾能力。深刻汲取“7·21”特大暴雨山洪泥石流灾害的教训，加强中心城区的防洪及雨洪调蓄，做好水系规划衔接，消除排水管道断点盲点，启动西郊砂石坑等蓄滞洪区建设。汛期前完成中心城20处下凹式立交桥积水治理，推进64处雨水泵站改造。严格落实新建项目和居住小区雨水控制利用规划，建成一批老旧小区雨水利用设施。加强高层建筑、人员密集场所等重点区域火灾防控，提高极端天气、地质灾害应对处置能力。细化各类灾害和突发事件应急预案，加强消防、防洪、防震、防疫等设施设备和队伍建设，完善重要物资紧急储备体系，健全快速响应机制。大力普及防灾减灾知识，积极开展体验教育和应急演练，建设200个综合减灾示范社区，提高市民防灾减灾意识和自救互救能力。

　　深入推进城市精细化管理。整合资源，全面推进网格化服务管理体系建设，加强城市管理综合执法，不断提升城市服务管理水平。探索建立地下管线综合管理体系，推进老旧管网升级改造，认真排查消除路面塌陷、管线破裂、燃气泄漏等安全隐患。研究制定雨污合流问题解决方案，完成50公里雨污管线改造。开展市容环境专项治理行动，推进240条重点大街、20片区域环境建设达标，抓好平房区、背街小巷等地区环境整治，消灭一批脏乱死角。加强户外广告、牌匾标识专项整治，完成二环内主要干道架空线入地任务。落实“门前三包”，建设整洁有序环境。推进信息技术在城市管理、公共服务等领域的广泛运用，让市民享受信息化、智能化建设成果。

　　(三)优化城市空间布局，促进城乡区域协调发展

　　深入落实主体功能区规划，推动首都功能核心区疏导并重、集约发展，城市功能拓展区重点开发、高端发展，城市发展新区有序开发、加快发展，生态涵养发展区适度开发、绿色发展，构筑全面协调可持续发展的城乡区域格局。

　　提升中心城区功能。实行最严格的建设规模控制，着力推进功能疏解，增强服务保障能力。完善9类专业市政场站及管网系统建设规划，抓紧解决市政设施不配套、标准低、管理责任不清等突出问题。充分利用区属资源，优化生活服务业网点布局，推广连锁化、品牌化、规模化经营。完善小区配套服务设施，打造宜居社区。坚持依法管理和升级改造相结合，逐步引导服装、建材、农产品等批发市场有序外移，推动大型长途客运站、医院康复服务机构等功能向郊区调整。做好群众身边的绿化美化，新增600公顷城市绿地，推进城市健康绿道和滨水绿廊工程，实施立体绿化，改造提升100条胡同街巷和100个老旧小区绿化景观，打造一批园林社区、景观街巷、绿色阳台。

　　加快城市副中心和新城建设。坚持聚焦通州，科学规划、分步实施，完善相关配套政策，按照“一核五区”布局，优化加快建设核心区起步区，力争重大基础设施、公共服务设施和重点项目建设取得较大突破，积极推动城市副中心建设。坚持以业兴城、职住平衡，加快顺义、亦庄-大兴、昌平、房山新城建设，打造功能完备、宜居宜业的综合性新城。同步推进门头沟、怀柔、平谷、密云、延庆等区域性新城建设，强化基础设施先行和特色产业支撑，带动区域城市化。要通过不懈努力，使城市副中心和新城的基础设施、公共服务、生态环境更具有吸引力，逐步发挥疏解中心城功能和人口的作用。

　　积极推动农村改革发展。创新体制机制政策，促进城乡要素平等交换和公共资源均衡配置，推动集体土地流转起来、资产经营起来、农民组织起来。总结推广乡镇统筹经验，因地制宜盘活农村集体建设用地，发展现代农村股份合作经济，让农民共享城市化过程中的土地增值收益。深化集体林权制度配套改革。创新农村金融服务。坚持科技兴农，完善现代农业服务体系，抓好国家现代农业科技城和示范区建设。健全农产品质量安全可追溯体系，加强外埠农产品基地建设，提高“菜篮子”保障水平。坚持以会兴业，做好世界种子大会、葡萄大会、月季大会、世界园艺博览会等筹备工作，推动都市型现代农业集约化、服务化发展。继续抓好新农村“三起来”工程，加大旧村改造力度，完成5万户农宅抗震节能改造，完善基础设施和公共服务设施运行管护长效机制。加快新型农村社区建设试点，推进村庄社区化管理。制定重点小城镇发展意见，鼓励大型企业与小城镇对接共建，切实增强小城镇综合承载能力。

　　不断缩小区域发展差距。实施第二阶段城南行动计划，全力办好第九届园博会，抓好石化新材料基地等重点项目，特别是要统筹安排，集中力量，完善专项规划，做好前期工作，扎实推进北京新机场和新航城建设。出台新首钢功能区发展意见，继续抓好永定河绿色生态发展带建设，促进西部转型发展。推进绿色北京示范区和雁栖湖生态发展示范区、密云生态商务区等重点项目建设，打造沟域经济品牌，启动山区1.4万人搬迁工作，推动生态涵养发展区绿色发展。认真解决整建制转居、超转人员社保资金等难题，加快城乡结合部地区产业调整改造，夯实城市功能拓展区、城市发展新区科学发展的基础。

　　(四)充分发挥全国文化中心示范作用，加快社会主义先进文化之都建设

　　深入实施文化创新战略，发挥文化引领风尚、服务社会、推动发展的作用，不断提升首都文化软实力。

　　加强社会主义核心价值体系建设。深入开展爱国主义、集体主义、社会主义教育，倡导富强、民主、文明、和谐，倡导自由、平等、公正、法治，倡导爱国、敬业、诚信、友善。大力弘扬爱国、创新、包容、厚德的北京精神，开展“做文明有礼的北京人”主题宣传实践活动，实施首都公民道德建设十大工程，深化公共文明引导行动。全面推进文明创建活动，广泛动员社会力量参与文明城市建设。抓好未成年人思想道德建设，加强大学生思想政治教育。落实新媒体发展战略，改进网络内容建设，唱响网上主旋律。

　　扎实推进重点文化惠民工程。加快建设北京文化活动中心等标志性设施，规划建设一批便民文体活动场所，推动重点镇文体活动中心建设，组织办好文艺大汇演等公益演出活动。创新文艺作品生产机制，鼓励创作高质量驻场演出剧目，提升文博会、艺术品产业博览会等重大活动举办水平，实施哲学社会科学和文化精品工程，推出更多原创、当代、北京的精品力作。加快建设覆盖城乡的全民健身公共服务体系，广泛开展群众体育活动。

　　增强文化创意产业竞争力。制定实施支持文化与科技、旅游等相关产业融合发展的意见，完善财税、金融等产业扶持政策。统筹用好100亿元文化发展专项资金，创新项目征集、资金使用机制，引导带动社会资本投资文化创意产业。加快建设中关村国家级文化与科技融合示范基地，加快发展设计创意、数字出版等融合型新业态，抓好国家广告产业园、出版创意产业园、首都核心演艺区、中国乐谷、怀柔影视基地等重大项目。推动国家版权交易中心、设计交易市场、北京文化产权交易所建设，完善文化投融资服务体系。启动第二批非时政类报刊出版单位转企改制，支持改制文艺院团建立现代企业制度，组建大型文化企业集团，鼓励民间资本参与国有文化企业改制重组。

　　大力传承优秀传统文化。加强历史文化名城整体保护，实施文物工作七大工程，加快钟鼓楼、地安门、前门东区等文保区修缮改造，继续推进中轴线、大运河申遗和“三山五园”历史文化景区建设，加强非物质文化遗产、地下文物保护利用，推动“老字号”振兴发展。建立市级投入机制，支持中心城区保护传统风貌，支持城市功能拓展区和发展新区建设对接安置房。创新保护利用模式和政策，积极吸引社会力量参与名人故居、胡同、四合院等修缮保护，合理开发利用具有市场前景的历史文化资源，充分彰显首都文化独特魅力。

　　(五)在改善民生和创新管理中加强社会建设，努力让人民群众过上更好生活

　　深入推进基本公共服务均等化，加大社会服务管理创新力度，继续办好惠民实事，不断增进人民群众福祉。

　　努力办好人民满意的教育。大力推进素质教育，促进义务教育均衡发展。落实中小学建设三年行动计划，推广学区教育资源共享、建立学校联盟等经验，支持30所城乡一体化学校建设。保障中小学生每天锻炼一小时，切实提高学生健康素质。实施名师名校长发展工程、农村教师素质提升工程，加大师资培养培训力度，鼓励优秀教师向薄弱学校流动。落实进城务工人员随迁子女参加中等职业学校考试录取政策，研究制定升学考试具体实施办法。完成学前教育三年行动计划，新增入园名额2.4万个。加快实施市属高校建设三年计划，加强重点学科和专业建设，支持高等院校内涵发展。开展职业教育分级制度改革，推进残疾人融合教育，完善终身教育体系，促进各级各类教育协调发展。

　　着力提高就业和居民收入水平。实施就业优先战略和更加积极的就业政策，制定城乡统一的用人单位招用补贴和绿色就业扶持政策，加大帮扶力度，完善以就业为导向的职业培训体系，鼓励以创业带动就业，实现城镇新增就业36万人，帮扶21万登记失业人员再就业和农村劳动力转移就业。健全劳动标准体系和劳动关系协调机制，推行工资集体协商，维护劳动关系和谐稳定。落实国家收入分配改革政策，制定实施居民收入倍增计划，建立工资正常增长机制，着力提升中低收入群体的收入水平。落实推进农村经济薄弱地区发展及低收入农户增收的意见，保持农民收入增速快于城镇居民。

　　进一步做好社会保障工作。加快实现社会保障人群全覆盖，稳步提高待遇水平。建立城乡居民大病医疗保险制度，深化总额预付等医保付费制度改革。推进城乡低保标准一体化，完善教育、医疗、住房等专项救助制度，推动社会救助从生存型向发展型转变。培育一批养老服务行业组织和龙头企业，大力发展康复型、护理型养老机构，新建养老床位1万张。坚持男女平等基本国策，提高妇女儿童的发展和权益保障水平。做好流浪未成年人救助保护工作。实施残疾人服务“一卡通”工程，加强残疾人社会保障和服务体系建设。加大住房保障力度，重点发展公租房、限价房，探索建立保障与市场相结合的住房供应体系，确保新建、收购16万套，竣工交用7万套保障房。完成1000万平方米老旧小区综合整治，抓好房山灾后永久性安置房建设，加快长辛店等五片棚户区改造。严格落实房屋安全质量责任制，抓好保障房配租配售和后期管理，完善公租房租金定价机制和分档补贴政策，减少承租家庭租金支出，让群众住得舒心、住得放心。

　　提升人民群众健康水平。扩大市级医院医药分开改革范围，做好区县医院改革试点，积极推进医疗服务价格调整。实施基层医疗机构综合改革，制定社会办医机构试点管理政策，鼓励引导社会资本参与公立医院改制重组。开展新一轮医疗机构药品集中采购，规范基本药物使用，加强药品监管，保障用药安全。大力开展医师规范化培训，加强社区卫生人员、乡村医生、中医医师的培养和使用。积极推进120急救体系城乡一体化建设，完善疾病预防控制体系，逐步构建公共卫生管理长效机制。启动天坛医院等大医院迁建工程，完成10个区域医疗中心一期建设，深入开展预约挂号、电子病历等便民服务，优化医疗资源配置和医疗服务体系。落实全民健康十年行动规划，加强健康教育，促进人民身心健康。

　　创新社会管理和维护首都稳定。进一步健全社会管理体制，深化基层社会管理和服务体系建设。建成500个“六型社区”，新建200个“一刻钟社区服务圈”，建设社区综合服务平台，初步实现城市社区基本公共服务全覆盖。引导社会组织健康有序发展，鼓励支持群众广泛参与社会服务管理。抓好社工队伍建设，加强志愿服务项目化管理，加快发展慈善事业。完善民族特色服务体系，发挥宗教界人士和信教群众在促进经济社会发展中的积极作用。加强全民国防教育和国防后备力量建设，健全优抚保障体系，进一步做好双拥共建工作，密切军政军民关系。建设军民融合科技创新示范基地，推动军民融合式发展。严格落实计划生育基本国策。加快研究北京市推行居住证制度的措施和办法，推进实有人口管理体系建设，创新服务管理，推动流动人口有序融入，促进首都人口长期均衡发展。加强村务公开，做好第九届村民委员会选举工作。深入开展“六五”普法，弘扬社会主义法治精神，打造公益律师等法律服务品牌，加快建设法治北京。加强公共安全体系和企业安全生产基础建设，开展标准化创建活动，依法打击非法违法生产经营建设、治理纠正违规违章行为，推进地下空间综合整治。加快社会信用体系建设。实施市场准入强制性标准，构建安全食品保障供给体系，严厉打击食品安全违法犯罪行为，让人民群众吃上放心的食品。进一步加强维稳和综合治理机制建设。坚持用群众工作统揽信访工作，实行重大决策社会稳定风险评估，完善多元调解机制，从源头上预防和减少社会矛盾。深入开展交通、治安、环境三大秩序整治，进一步强化社会面防控，统筹推进“平安北京”建设，努力营造和谐稳定的社会环境。

　　(六)加强生态文明建设，为人民群众创造良好生产生活环境

　　坚持节约优先、保护优先、自然恢复为主的方针，尊重自然、顺应自然、保护自然，建立体现生态文明要求的目标体系、考核办法，加快建设天蓝、地绿、水净的美丽城市。

　　深入推进资源节约和减排降耗。落实最严格的耕地保护制度，加强土地节约集约利用评价。继续抓好重点领域节能减排，健全落后产能退出机制，推进东方化工厂关停搬迁，支持重点污染企业实施清洁生产。推行新建住宅75%节能标准，实施既有建筑节能改造1000万平方米，完成住宅供热计量改造6500万平方米。促进合同能源管理和节能服务产业发展，启动一批重点用能单位节能低碳改造，全面完成市级政府部门节能改造。加快调整能源结构，提高能源综合利用效率，建成大唐煤制气一期工程，实现四大热电中心主体竣工，开工建设陕京四线，实施新能源高端示范项目，加快推进能源应急储备设施建设。积极推广应用节能新技术、新产品，倡导绿色低碳的生产生活方式。

　　增强水资源水环境承载能力。坚持量水而行、以供定需，实行最严格的水资源管理制度。加快推进南水北调及配套工程建设，完成第三水厂、第八水厂改扩建工程，新建改造520公里供水管网，确保2014年长江水调得进、用得上。做好应急水源地的运行保障。加快污水处理和再生水利用工程建设，全市污水处理率达到84%，再生水利用量达到8亿立方米。继续推进三大水系综合治理，整治280公里中小河道，抓好水毁河道修复、小水库除险加固，建成34条生态清洁小流域，提升中小河道的防洪排涝能力。

　　深化以大气为重点的污染治理。严格落实2012-2020年大气污染治理措施，加大PM2.5治理力度，切实做好压煤、换车、降尘等工作，确保空气中主要污染物浓度平均下降2%。淘汰18万辆老旧机动车，在公交、环卫、政府机关推广使用新能源汽车。完成城区1600蒸吨燃煤锅炉、4.4万户平房采暖清洁能源改造，基本实现中心城区“无煤化”。加快农村清洁能源改造。严格管控施工现场扬尘，抓好绿色施工和道路清扫保洁。加强空气质量监测分析和信息发布，推进区域联防联控。按照最先进的技术、最严格的标准，加快垃圾焚烧、生化处理等设施建设。完善生活垃圾全过程管理体系，推动餐厨垃圾就地资源化处理，居住小区垃圾分类达标率提高到60%。启动高碑店等污泥处置工程，推进电器电子废弃物、建筑垃圾等资源化处置和回收利用，促进减量化、资源化、无害化。

　　着力抓好森林绿地建设。继续实施平原百万亩造林工程，确保全年完成35万亩任务。加快建设南中轴等森林公园，新增万亩城市森林10处。广泛动员社会力量参与，探索建立碳汇交易机制，倡导企业积极履行社会责任，认建认养绿地。研究完善养护管理的长效机制，科学规划、合理发展林下经济。全面启动京津风沙源治理二期、三北防护林五期工程，继续抓好低效林改造、中幼林抚育等工作，加强生物多样性保护，做好山区水土保持工作，增强山区生态屏障功能。

　　(七)坚定不移深化改革，提高开放型经济发展水平

　　进一步解放思想，坚决破除一切妨碍科学发展的思想观念和体制机制弊端，不断拓展对外开放的新空间、新领域，为首都科学发展增添新的动力和活力。

　　深化重要领域改革。抓住经济社会发展中的突出矛盾和问题，遵循经济规律，正确处理政府与市场的关系，及时推出改革举措。一是深化国有企业改革。加快国有资产证券化步伐，继续推进国有企业改制重组，支持与各类所有制企业深度合作，集中优势资源投向战略性重点领域，培育具有核心竞争力的大企业集团。二是深化投资体制改革。出台多个领域民间投资实施细则，切实放宽市场准入，完善投资回报机制，打破“弹簧门”、“玻璃门”等体制障碍，推出一批公共领域市场化建设试点项目。三是深化财税体制改革。完善市对区县财政转移支付政策，深入实施预算绩效管理，提高财政资金特别是大额专项资金的使用效益。推进税收征管改革，抓好“营改增”试点改革。做好第三次经济普查工作。四是稳步理顺价格体系。适时推进阶梯水价、非居民生活垃圾收费等改革，引导资源合理配置。

　　培育开放发展新优势。稳定扩大外贸出口，积极发展外贸转型升级示范基地，支持拥有自主品牌、自主知识产权企业和中小企业开拓国际市场，培育汽车、医疗器械等新的出口增长点，鼓励对外投资带动出口。加快推进天竺综保区、平谷国际陆港、通州口岸建设，大力发展口岸经济。全力办好第二届“京交会”。支持服务外包加快发展，促进文化创意、软件等新兴服务贸易增长。加强投资促进工作，提高利用外资综合优势和总体效益，实现引资、引技、引智有机结合。做好企业“走出去”服务，鼓励企业参与国际展会、创制国际标准和境外并购。高水平办好大型国际会议和会展活动。继续扩大与港澳地区的经贸往来与合作，做好对台和侨务工作。

　　更好地服务区域发展。加强与周边省区市的合作，加快推进首都经济圈建设，共同推动区域一体化进程。深入落实合作框架协议，推进以大气污染防治、水资源保护利用为重点的区域生态环境建设，探索与周边地区共建产业基地的途径和模式，促进基础设施互联互通和城镇、产业体系协调发展。发挥教育、科技、人才、市场优势，高水平做好援藏、援疆、援青工作，不断深化与对口支援地区的经济技术合作。

　　三、努力建设人民满意的服务型政府

　　面对新形势、新任务，各级政府及其部门要坚持以人为本、执政为民，增强忧患意识、创新意识、宗旨意识和使命意识，深入开展以为民务实清廉为主要内容的群众路线教育实践活动，进一步转变职能、改进作风、优化服务，不断提升政府工作效率和水平。

　　深入学习贯彻党的十八大精神。要紧密联系实际，推动学习贯彻走向深入，坚定中国特色社会主义的道路自信、理论自信、制度自信，切实把思想统一到党的十八大精神上来，把力量凝聚到实现党的十八大确定的目标任务上来。要把学习的成果体现到加强政府自身建设、履行好政府职责中，体现到完成好今年各项任务、全力推动首都科学发展中，始终保持昂扬向上的精神状态，坚定不移推进中国特色社会主义在北京的实践。

　　着力转变政府职能。深化行政管理体制改革，积极探索行政执法资源的优化整合。做好第六批行政审批事项清理工作。加快市级政务服务中心建设，优化行政审批流程，更好地服务基层、企业和群众。完善事业单位分类改革配套政策，推动一批经营类事业单位转企改制，支持公益类事业单位发展。全面正确履行政府职能，把工作重心和主要精力更多地转向创造良好发展环境、提供优质公共服务和维护社会公平正义上来。坚持为民、利民、便民，扎实做好服务群众工作。创新和改进服务管理方式，大力发展电子政务，健全部门协调配合机制，进一步理顺部门关系。加强政府绩效管理，不断提高行政效率和服务水平。

　　着力推进依法行政。模范遵守和执行各项法律法规，严格按照法定权限和程序行使权力，认真落实市人大及其常委会的决议和决定，加强与市政协的工作沟通和协商，认真听取各民主党派、工商联、无党派人士和人民团体的意见，自觉接受人民群众和新闻舆论监督。健全决策机制和程序，建立问责和纠错制度，提高决策科学化、民主化、法制化水平。高水平做好政府规章、规范性文件制定工作，构建行政执法平台，加强和改进行政执法，加快建设法治政府。大力推进政务公开，切实提高政府的公信力。

　　着力加强政风建设。坚决贯彻落实中央“八项规定”和我市15条具体措施，进一步改进工作作风，密切联系群众。在乡村、企业、社区建立联系点，轻车简从深入基层调查研究，切实解决发展难题。大力精简会议活动，下决心改进会风文风。坚持勤俭办一切事业，严格控制“三公经费”，狠刹奢侈浪费之风，节省资金多办打基础、惠民生的实事好事。加强督查督办，各项工作都要有部署、有督促、有落实，有检查、有奖惩、有必要的事后评估。加强公务员教育培训和管理，建设一支高素质的公务员队伍。各级领导干部要以身作则、率先垂范，要求别人做到的自己首先要做到，要求别人不做的自己坚决不做，以实际行动践行全心全意为人民服务的根本宗旨。

　　着力加强廉政建设。认真贯彻落实十八届中央纪委二次全会精神，坚持常抓不懈，坚定不移把反腐倡廉建设引向深入。制定五年惩防体系建设实施意见，深入推进廉政风险防控管理，全面落实廉政建设责任制，加大从源头上预防和治理腐败力度。严格落实行政问责制度，加强效能监察，整治庸懒散奢等不良风气。加强经济责任审计，强化审计监督。坚决纠正部门和行业的不正之风，继续加大查办案件工作力度，着力解决发生在群众身边的腐败问题，切实做到从严治政、队伍清廉、政府清明。

　　各位代表！首都发展已经站在新的历史起点上，梦想孕育奇迹，万事出自艰辛。空谈误国，实干兴邦。让我们紧密地团结在以习近平同志为总书记的党中央周围，高举中国特色社会主义伟大旗帜，坚持以邓小平理论、“三个代表”重要思想、科学发展观为指导，在中共北京市委坚强领导下，解放思想，改革开放，凝聚力量，攻坚克难，深入推进中国特色世界城市建设，为实现中华民族伟大复兴作出新的更大贡献。
